# Mr. Keetley's Paper on the Advantages of Appendicostomy

**Published:** 1909-01-30

**Authors:** 


### SURGERY.

### MR. KEETLEY'S PAPER ON THE ADVANTAGES OF APPENDICOSTOMY.

On November 10, 1908, Mr. Keetley read a paper before the Royal Society of Medicine dealing with his experience of appendicostomy, appendicotomy, and of a third operation which he calls "appendix transplantation." The original character of the opinions he expressed therein has given rise to criticism in many quarters, as all original views are bound to do. The particular feature of his paper which has called forth so much comment is his expressed opinion that in many cases of appendicitis, even acute ones, he regards it as better to fix the appendix in the muscles of the abdominal wall than to remove it completely.

At the date of his paper appendicostomy had already been advocated and practised for a variety of conditions. Chief among these, of course, were the different forms of colitis. But it had also been employed for constipation, ileus due to toxæmia, intestinal hæmorrhage, and in intussusception to prevent recurrence. He then explained that the special object of his paper was to prove "(1) that transplantation of the appendix vermiformis so that the whole or the greater part of it from its root in the cæcum lies permanently embedded in the abdominal wall, would produce the good results of excising it; (2) that it is practicable and safe; and (3) that transplantation should in many cases be preferred to appendicectomy."

He contended that it is unjust to regard the appendix as a vestigial and retrogressive organ as is commonly done, and referred to the work of Sir William MacEwen, who demonstrated that the appendix has functions which are possibly of great value, and to Metchnikoff's hypothesis that the degenerations of old age are in part at any rate, due to toxins formed by the bacteria which inhabit the large intestine. He explained that inflammatory changes in the appendix are not in themselves dangerous; they constitute only a menace to the life of the patient by reason of the intimate relation of the appendix to the peritoneum and of the concomitant peritonitis. It is, therefore, fair to argue that if the appendix were artificially moored in less inflammable surroundings the danger would be reduced to a minimum. To quote his own expressive phrase, in Mr. Keetley's opinion "an appendix transplanted is an appendix disarmed." To prove the truth of this he quoted the case of an elderly lady who had had her appendix transplanted some time previously on account of obstruction. A fish bone perforated the mucous membrane of the appendix. An abscess in the abdominal wall resulted. This was opened and the trouble subsided in a day or two. He described the technique of the operation as he performed it, and he laid stress on the following points. It is important to avoid tension, and to do this it is sometimes necessary to divide adhesions; the meso-appendix which carries the blood supply must not be damaged or constricted. The appendix should be arranged so as to lie in the abdominal wall with its apex upwards and outwards and brought out through a separate buttonhole incision. It is better not to open the appendix until it is adherent in its new position, and if a catheter must be left in, a small one should be employed—a large one may press on the appendix and thus disturb its nutrition. Finally he described an ingenious method of dealing with the apex of the appendix, if it should be desired to keep it open for any length of time. The whole of the sero-muscular coat of the protruding portion is removed, and the remaining portion of the mucous coat is turned back on itself cuff-wise, so that a little nipple results, the orifice of which can always be found.

With regard to obstinate constipation he thinks that surgical interference is sometimes necessary, and appendicostomy is the best of all the operative procedures which have so far been devised for this condition. His experience of it has been most satisfactory. With regard to appendicitis he made the reservation that all cases are not suitable for transplantation, and referred to his Cavendish lecture in which he had dealt with that point. At that time he had pointed out that the following cases are not suitable: (1) Appendices which are obliterated, tuberculous, actinomycotic, or gangrenous; (2) appendices gangrenous or perforated near the proximal end; and (3) those which can not be fixed in the abdominal wall without causing undue tension or the risk of interfering with their blood supply in the meso-appendix.

Mr. Keetley's experiences are exceedingly interesting, but it is difficult to accept his contentions without demur. If, for instance, the appendix is not really a vestigial organ, we should expect that patients in whom it has been removed for appendicitis would afterwards suffer some inconvenience from its absence. Yet this is rarely if ever found to be the case; and it does not seem a sufficiently strong argument to say that the patient is really the poorer because he may subsequently want his appendix in order to have an appendicostomy performed say for colitis or for constipation. We find ourselves in sympathy with the surgeon (quoted by Mr. Keetley) who declared that he would sooner have his appendix in his pocket than in his abdominal wall. In fact it comes as somewhat of a shock when we had fondly imagined that we had evolved in appendicectomy an operation as nearly ideal as any surgical procedure can be, to be suddenly told that it is not the best operation after all.

With regard to constipation we are of opinion that operative proceedings are rarely if ever justifiable. Efficient treatment with purges and enemata nearly always succeeds if persisted in; and it is difficult to see why enemata introduced *per appendicem* should have more virtue than rectal injections. But if any operation is ever justifiable for this condition we must admit that we would prefer appendicostomy to the more drastic measures such as excision of the colon, which have been advocated. In colitis proper, however, we believe that appendicostomy is a most valuable measure.

